# Prevalence, awareness, and treatment of anemia in Chinese patients with nondialysis chronic kidney disease: First multicenter, cross-sectional study

**DOI:** 10.1097/MD.0000000000003872

**Published:** 2016-06-17

**Authors:** Ya Li, Hao Shi, Wei-Ming Wang, Ai Peng, Geng-Ru Jiang, Jin-Yuan Zhang, Zhao-Hui Ni, Li-Qun He, Jian-Ying Niu, Nian-Song Wang, Chang-Lin Mei, Xu-Dong Xu, Zhi-Yong Guo, Wei-Jie Yuan, Hai-Dong Yan, Yue-Yi Deng, Chen Yu, Jun Cen, Yun Zhang, Nan Chen

**Affiliations:** a Department of Nephrology, Ruijin Hospital, School of Medicine, Shanghai Jiao Tong University; b Department of Nephrology and Rheumatology, Shanghai Tenth People's Hospital, School of Medicine, Tongji University; c Department of Nephrology, Xinhua Hospital, School of Medicine, Shanghai Jiao Tong University; d Department of Nephrology, The 455th Hospital of PLA; e Department of Nephrology, Renji Hospital, School of Medicine, Shanghai Jiao Tong University; f Department of Nephrology, Shuguang Hospital, Shanghai University of Traditional Chinese Medicine; g Department of Nephrology, Shanghai Fifth People's Hospital, Fudan University; h Department of Nephrology, Shanghai Jiao Tong University Affiliated Sixth People's Hospital; i Department of Nephrology, Shanghai Changzheng Hospital, Second Military Medical University; j Department of Nephrology, Shanghai Minhang District Central Hospital; k Department of Nephrology, Shanghai Changhai Hospital, Second Military Medical University; l Department of Nephrology, Shanghai Jiao Tong University Affiliated First People's Hospital; m Department of Nephrology, Shanghai East Hospital, Tongji University School of Medicine; n Department of Nephrology, Longhua Hospital, Shanghai University of Traditional Chinese Medicine; o Department of Nephrology, Shanghai Tongji Hospital, Tongji University School of Medicine; p Department of Nephrology, Shanghai Jiangong Hospital; q Department of Nephrology, Shanghai Yangpu District Central Hospital, Shanghai, China.

**Keywords:** anemia, awareness, chronic kidney disease, diabetic nephropathy, prevalence

## Abstract

This was the first multicenter, cross-sectional survey to assess the prevalence of anemia, patient awareness, and treatment status in China. Data of patients with chronic kidney disease (CKD; age, 18–75 years; both out- and inpatients) from 25 hospitals in Shanghai, seeking medical treatment at the nephrology department, were collected between July 1, 2012 and August 31, 2012. The prevalence, awareness, and treatment of anemia in patients with nondialysis CKD (ND-CKD) were assessed. Anemia was defined as serum hemoglobin (Hb) levels ≤12 g/dL in women and ≤13 g/dL in men. A total of 2420 patients with ND-CKD were included. Anemia was established in 1246 (51.5%) patients: 639 (51.3%) men and 607 (48.7%) women. The prevalence of anemia increased with advancing CKD stage (χ^2^_trend_ = 675.14, *P* < 0.001). Anemia was more prevalent in patients with diabetic nephropathy (68.0%) than in patients with hypertensive renal damage (56.6%) or chronic glomerulonephritis (46.1%, both *P* < 0.001). Only 39.8% of the anemic patients received treatment with erythropoietin and 27.1% patients received iron products; furthermore, 22.7% of the patients started receiving treatment when their Hb level reached 7 g/dL. The target-achieving rate (Hb at 11–12 g/dL) was only 8.2%. Of the 1246 anemia patients, only 7.5% received more effective and recommended intravenous supplementation. Anemia is highly prevalent in patients with ND-CKD in China, with a low target-achieving rate and poor treatment patterns. The study highlights the need to improve multiple aspects of CKD management to delay the progression of renal failure.

## 1 Introduction

Chronic kidney disease (CKD) is a life-threatening, multifactorial disorder with a continuously increasing trend worldwide.^[1–3]^ An inevitable and frequent complication of CKD is anemia. The complications of anemia are neurocognitive impairment, sleep disturbances, CKD progression, cardiovascular comorbidities, and higher mortality.^[4–9]^ Anemia correlates strongly with both CKD progression and patient survival.^[10]^ Furthermore, anemia in patients with CKD has a direct effect on healthcare costs and quality of life (QoL) during both predialysis and dialysis stages.^[4,6,7,9,11]^ The prevalence of anemia (with or without CKD) significantly increases with advancing age.^[8]^ The causes of anemia in patients with CKD include erythropoietin (EPO) deficiency, decreased responsiveness to EPO, shortened red blood cell survival, iron deficiency, and chronic inflammation.

Several clinical practice guidelines such as the National Kidney Foundation Kidney Disease Outcomes Quality Initiative (NKF-KDOQI),^[11–13]^ the European Best Practice Guidelines,^[14]^ and the Kidney Disease Improving Global Outcomes^[15]^ recommend management of anemia as an integral part of CKD management. For practical reasons, anemia has been studied in patients with CKD receiving dialysis. However, anemia is increasingly diagnosed in patients with early and mid-stage CKD.^[16,17]^

The cross-sectional estimate of the overall CKD prevalence in China is approximately 10.8% (119.5 million).^[18]^ Evidence suggests that treatment of anemia in the predialysis and dialysis populations remains suboptimal.^[19–21]^ Treatment strategies for anemia in CKD include use of erythropoiesis-stimulating agents (ESAs), iron therapy, ESA resistance, and blood transfusion.^[13]^ Data from the Dialysis Outcomes and Practice Pattern Study (DOPPS) and Shanghai Renal Registry 2011 indicated that a substantial proportion of the patients did not achieve the recommended hemoglobin (Hb) targets in China and that the use of intravenous iron was also less. Only 39% to 46% of the patients who underwent hemodialysis achieved a target Hb of 10 to 12 g/dL (similar to the target recommended by the NKF-KDOQI at 11–12 g/dL).^[22]^

In contrast to the relatively abundant information regarding anemia in patients with CKD on dialysis worldwide,^[8]^ a particularly significant unmet need of prevalence and treatment of anemia in patients with nondialysis CKD (ND-CKD) continues to persist in China. Because the spectrum of the underlying diseases in China differs from that in western countries, it is necessary to examine the prevalence of anemia in nondialysis patients in China.

Because of the ever-growing population of patients with CKD, nephrologists are concerned about anemia in patients with ND-CKD and the treatment modalities to delay end-stage renal disease (ESRD) and reduce the high cardiovascular risk associated with it.^[23,24]^ Furthermore, reliable prevalence data on anemia in ND-CKD are essential to develop national and international health policies for the prevention and control of ND-CKD.

This first multicenter, cross-sectional study was therefore conducted to examine the prevalence of anemia, patient awareness, and treatment status in patients with ND-CKD in Shanghai, a highly developed region in China.

## 2 Methods

### 2.1 Study design and participants

This first multicenter, cross-sectional, noninterventional study included data from 25 Shanghai hospitals (secondary or tertiary) that were selected using a stratified random sampling method based on government-sponsored hospital rating and geographical distribution (ChiCTR; Trial registration number: ChiCTR-ECS-12002383). The participating centers were included if they had an outpatient clinic dedicated to the care of patients with ND-CKD (not receiving dialysis or transplantation), with the attending patient population seen at least twice a year, and if ≥500 patients were regularly followed up in the clinic.

The study comprised adult patients with ND-CKD (age, 18–75 years; both out- and inpatients) who presented at the nephrology department of the included hospitals for seeking medical advice/treatment between July 1, 2012 and August 31, 2012. One nephrologist in each center underwent formal training for data entry. All participants were voluntary and submitted written informed consent. Patient identity was kept anonymous. Each participating site procured approval from the institutional review board before study initiation. Patients were excluded from the study if they were pregnant or lactating; had renal transplantation; or were comorbid with severe digestive tract disorders, including active ulcer in the digestive tract, chronic hemorrhagic gastritis, chronic hepatitis B, decompensated hepatic dysfunction, and active hepatitis. In addition, patients’ comorbid with severe hematological disorders (e.g., leukemia, aplastic anemia, lymphoma, myelodysplastic syndrome, multiple myeloma, and hemolytic anemia); those reporting active malignancy or severe gynecological hemorrhagic disorders, cirrhosis and advanced heart failure (NYHA IV); or those with secondary nephritis such as active lupus nephritis or antineutrophil cytoplasmic autoantibodies-associated vasculitis were excluded. All the participating centers used the same criteria to define existing comorbidities and pathologies.

### 2.2 Definition of anemia

The diagnosis of anemia was made according to the 2001 World Health Organization (WHO) diagnostic criteria. Anemia was defined as a Hb of ≤13.0 g/dL in men and ≤12.0 g/dL in women, as recommended by the National Anemia Action Council and the WHO. Calculation of the target-achieving rate was based on the recommendations of the 2007 NKF-KDOQI, with a target range >11 g/dL and not exceeding 12 g/dL regardless of the comorbidities or dialysis status.^[25]^

### 2.3 Patient analysis and data collection

Data regarding the demographics and medical history were collected by physicians based on a custom-designed questionnaire, including sex, age, height, body weight, body mass index, occupation, and blood pressure, between July 1, 2012 and August 31, 2012. Laboratory examinations included routine blood tests, urine tests, serum creatinine (SCr), iron, ferritin, and transferrin saturation. The underlying disease (e.g., hypertension, diabetes, and glomerulonephritis) was recorded as diagnosed by the treating physician. Hypertension was diagnosed in patients on the basis of the cutoff recommended by the guidelines issued jointly by the WHO and the International Society of Hypertension—systolic blood pressure ≥140 mm Hg and/or diastolic blood pressure ≥90 mm Hg in adults aged 18 years or older not taking antihypertensive agents, regularly using antihypertensive drugs or any use of antihypertensive medication in the past 2 weeks regardless of blood pressure, or a history of hypertension.^[26]^ Diabetes mellitus was defined as requiring antidiabetic drugs or meeting the diagnostic criteria for diabetes mellitus specified by the 1999 WHO and the Chinese Guideline for Diabetes Prevention and Treatment (characteristic symptoms and casual blood glucose, ≥11.1 mmol/L; fasting blood glucose, ≥7 mmol/L; or 2-hour oral glucose tolerance test, ≥11.1 mmol/L).^[27]^ CKD was classified and defined as per the NKF-KDOQI recommendations, which was based on kidney damage with or without a decrease in the glomerular filtration rate (GFR). Participants were stratified into 5 stages based on GFR values: stage 1 (≥90 mL/min/1.73 m^2^), stage 2 (60–89 mL/min/1.73 m^2^), stage 3 (30–59 mL/min/1.73 m^2^), stage 4 (15–29 mL/min/1.73 m^2^), and stage 5 (<15 mL/min/1.73 m^2^). GFR was calculated using a simplified Modification of Diet in Renal Disease equation as follows^[28,29]^: 
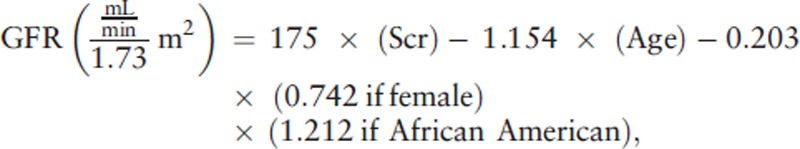


where SCr is in mg/dL and age is in years.

We reviewed medical history of subjects during study. The patients who had anemia history and had received treatment were classified as having anemia with normal Hb. Patients were enquired regarding anemia history, the treatment which they were receiving (EPO or iron products), time of initiation, and the route of treatment. The following data were collected in subjects receiving EPO treatment which included Hb level, weekly EPO dosage, and treatment route (subcutaneous vs intravenous). Patients receiving iron products were evaluated for baseline serum iron, ferritin, and transferrin saturation, as well as the treatment route (intravenous vs oral).

Awareness rate of anemia in ND-CKD patients represents the percentage of patients who were of anemia at the time of the survey. All completed questionnaires were reviewed by principal investigators at each of the participating study sites.

### 2.4 Statistical analysis

Both medical recorder and laboratory testing results were double-entered in EpiData 3.1 (Build 270108; The EpiData Association, Odense, Denmark). Continuous variables were expressed as mean ± standard deviation or median ± interquartile range (IQR). Categorical variables were described as numbers or proportions and analyzed across CKD stages and varying background diseases using χ^2^ test. All statistical analyses were performed using SAS 9.3 (SAS Institute, Inc, Cary, NC). A 2-sided *P* ≤ 0.05 was considered statistically significant for all analyses.

## 3 Results

### 3.1 Patient characteristics

A total of 2420 patients with ND-CKD (men, 1297 [53.6%]; women, 1123 [46.4%]) with a mean age of 54.51 ± 16.95 years were enrolled. CKD staging included: n = 642 for stage 1, n = 440 for stage 2, n = 509 for stage 3, n = 341 for stage 4, and n = 488 for stage 5. Other baseline characteristics of the participants are shown in Table 1. A large proportion of the population (n = 509) had stage 3 CKD (30–59 mL/min/1.73 m^2^). A majority of the patients (n = 293, 60.8%) having hypertension belonged to stage 5 CKD.

**Table 1:**
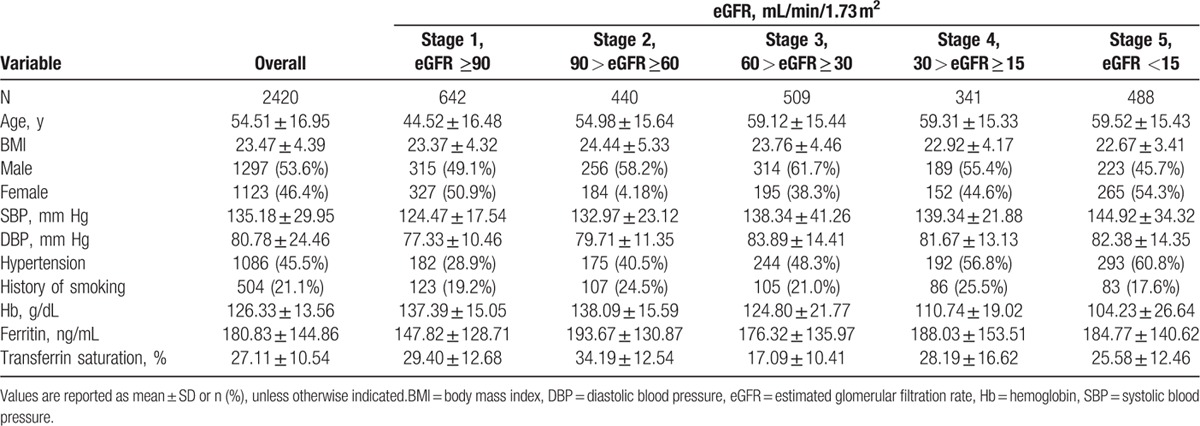
Demographic and clinical characteristics of the subjects.

### 3.2 Prevalence of anemia

**Figure 1:**
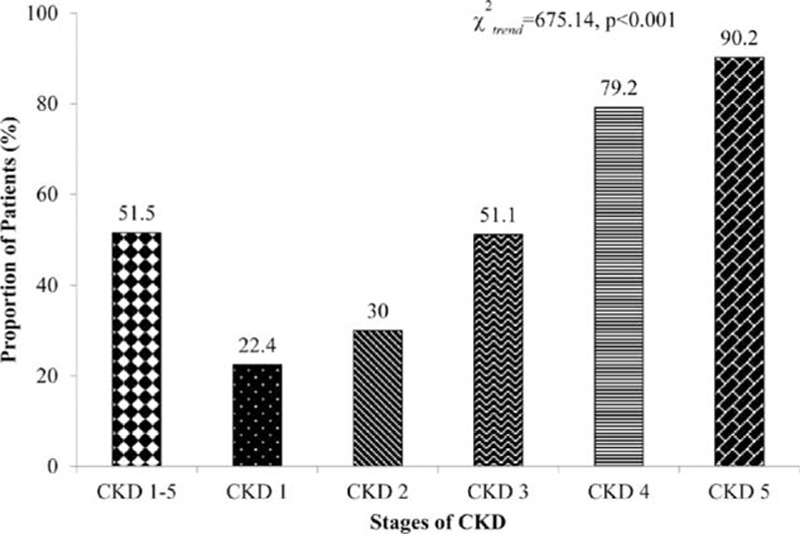
Prevalence rate of anemia.

The prevalence of anemia was 51.5% in the overall study sample, with 639 (51.3%) men and 607 (48.7%) women. An increasing trend of anemia was associated with advancing CKD stage (Fig. 1), that is, 22.4% in stage 1, 30.0% in stage 2, 51.1% in stage 3, 79.2% in stage 4, and 90.2% in stage 5 (χ^2^_trend_ = 675.14, *P* < 0.001).

### 3.3 Awareness of anemia

**Figure 2:**
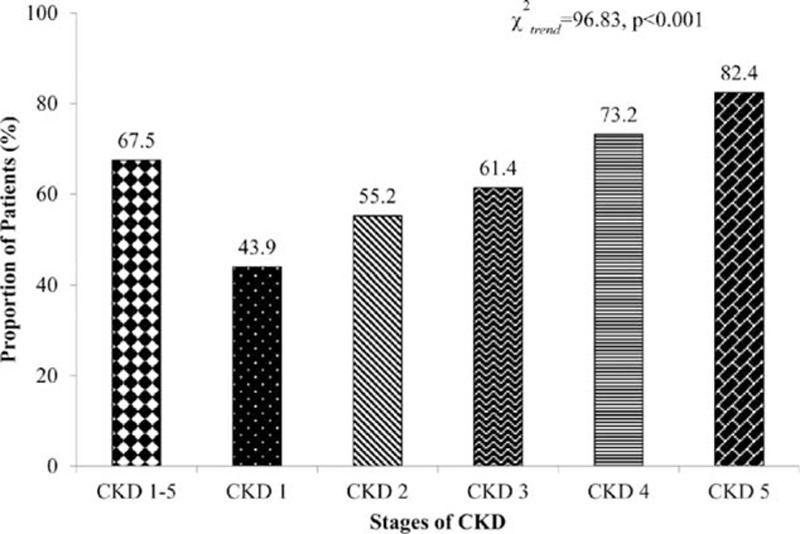
Awareness rate of anemia.

Anemia awareness was 67.5% in the overall sample. An increasing trend of anemia awareness was observed with advancing CKD stage (Fig. 2), that is, 43.9% in stage 1, 55.2% in stage 2, 61.4% in stage 3, 73.2% in stage 4, and 82.4% in stage 5 (χ^2^_trend_ = 96.83, *P* < 0.001).

### 3.4 Anemia versus underlying diseases

**Figure 3:**
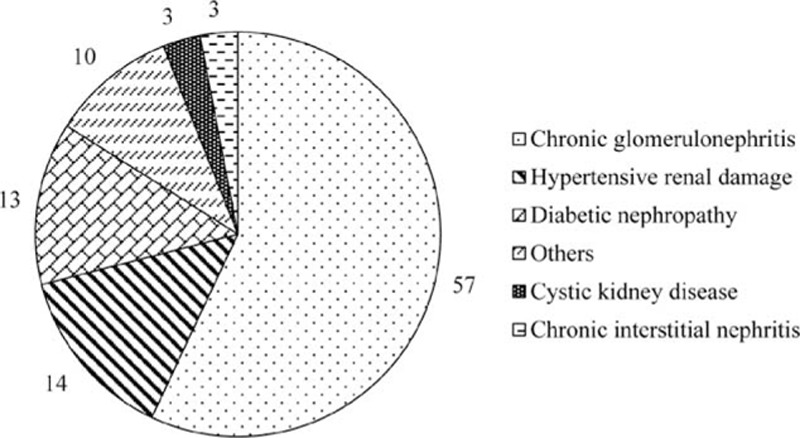
Common underlying diseases.

The most common underlying disease was chronic glomerulonephritis (n = 1301, 57.26%), followed by hypertensive renal damage (n = 318, 13.69%) and diabetic nephropathy (n = 303, 13.34%) (Fig. 3). Overall, anemia was more common in patients with diabetic nephropathy (68.0%) than in patients with hypertensive renal damage (56.6%) or chronic glomerulonephritis (46.1%, all *P* < 0.001). At stage 1, the prevalence of anemia was comparable across varying underlying diseases (diabetic nephropathy group, 25.0%; hypertensive renal damage group, 21.4%; chronic glomerulonephritis group, 22.4%); however, these rates started to diverge at stage 2. An increasing trend of anemia was observed with advancing CKD stage (Table 2). The prevalence of anemia was particularly high in patients with diabetic nephropathy.

**Table 2:**
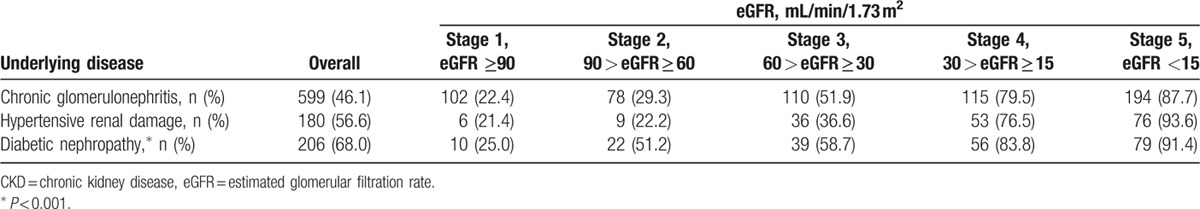
Association between anemia and primary etiology of CKD.

### 3.5 Anemia treatment

Of the 1246 CKD patients with anemia, 559 (44.9%) patients reported being treated for it. The mainstay of treatment for anemia in patients with CKD is use of either EPO or iron products. The treatment rate of anemia significantly increased with advancing CKD stage. The percentage of patients receiving anemia treatment was 19.4% in stage 1, 11.4% in stage 2, 26.9% in stage 3, 46.3% in stage 4, and 73.0% in stage 5 (*P* < 0.001). The Hb target of 11 to 12 g/dL was achieved in 8.2% of the 559 patients receiving treatment and 26.4% considering 10 to 12 g/dL as the target. This rate was found to increase from CKD stage 1 to CKD stage 3 which then demonstrated a decreasing trend till stage 5: 10.7% in stage 1, 13.3% in stage 2, 17.1% in stage 3, 8.8% in stage 4, and 5.6% in stage 5 (Table 3).

**Table 3:**
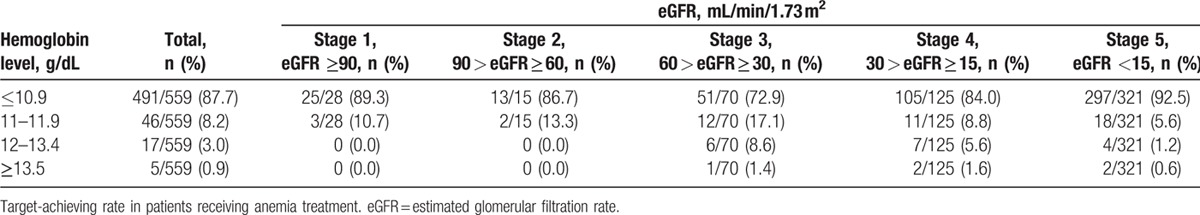
Anemia control.

#### 3.5.1 EPO treatment

**Figure 4:**
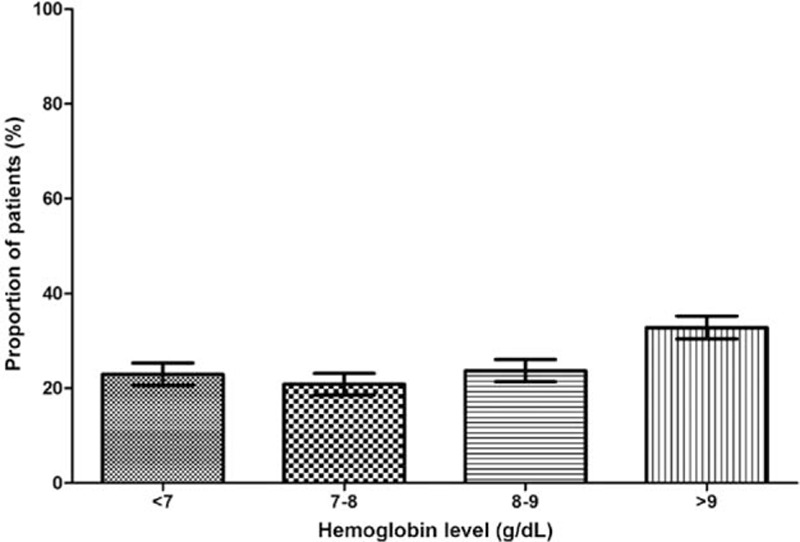
Hb levels of erythropoietin treatment initiation.

Of the 1246 patients with anemia, 496 (39.8%) received EPO treatment (493 received EPO-α). The Hb level in these patients was found to be 9.2 g/dL (IQR: 8.0–11.0 g/dL); Hb level was 7.0 to 8.0 g/dL in 20.5% of the patients and <7.0 g/dL in 22.7% of the patients (Fig. 4). The rate of patients with Hb level <10 g/dL was 58.3% (726/1246) in the overall study sample of anemic patients.

### 3.6 Treatment with iron products

Of the 1246 patients with anemia, 338 (27.1%) patients received iron products. Of these patients, 29.7% reported ferritin levels at ≤100 ng/mL, and 41.0% had ≤20% transferrin saturation. The ferritin level (≤100 ng/mL) decreased gradually as the CKD stage advanced. Transferrin saturation ≤20% was highest in patients with stage 5 CKD (stage 1: 41.2%; stage 2: 20.0%; stage 3: 42.3%; stage 4: 36.2%; stage 5: 43.4%). The association between renal function and iron stores in patients with CKD is shown in detail in Table 4. A majority of the patients received oral formulation (n = 245/1246; 19.7%), whereas 7.5% of the patients received intravenous supplementation.

**Table 4:**
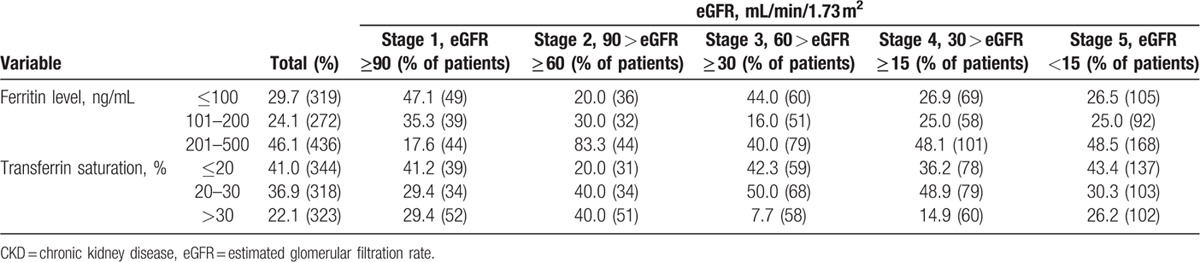
Association between renal function and iron stores in patients with CKD.

## 4 Discussion

Anemia increases the risk of dialysis in patients with CKD and correlates significantly with the mortality and hospitalization rate in patients undergoing dialysis.^[30,31]^ Nephrologists are concerned about anemia in patients with ND-CKD as it seems to be the main independent modifiable risk factor of cardiovascular and renal damage. Moreover, there are more number of patients with ND-CKD (patients with CKD before receiving dialysis) than patients receiving regular dialysis. In such a scenario, reliable prevalence data are crucial to develop health policies for early diagnosis, prevention, and increasing ND-CKD awareness.

Few previous studies conducted in China have demonstrated an increasing prevalence of CKD in certain provinces and municipalities (Beijing, Taian, Zhejiang and Guangdong, and Tibet); however, these studies do not provide insights into the epidemiology of CKD in association with anemia.^[32–35]^ To date, no other studies have focused on the epidemiology of anemia in patients with ND-CKD in China. This was the first multicenter, cross-sectional, noninterventional study to examine the prevalence of anemia, patient awareness, and treatment status in Shanghai, a highly developed region in China.

The findings of this study revealed a high prevalence of anemia (51.5%) in patients with ND-CKD in China, with a rate as high as 22.1% even in stage 1 disease. Therefore, treatment of anemia could be significantly beneficial at both patient and social levels. The prevalence of anemia increased with advancing CKD stage. In the current study, the prevalence of anemia was >90% in patients with stage 5 CKD; this may be attributed to the decreased production of endogenous EPO due to a decrease in eGFR, as well as metabolic disturbances such as uremic toxins and electrolyte and acid-base imbalances.

The results from the third community-based National Health and Nutrition Examination Survey (NHANES III) (1988–1994) revealed the prevalence of anemia to be <2% in patients with GFR >60 mL/min and also concluded lower kidney function has strong correlation with higher anemia prevalence among the US adult population. The prevalence of anemia (Hb level <12 g/dL in men and <11 g/dL in women) showed an increase from 1% (95% confidence interval [CI], 0.7%–2%) at GFR of 60 mL/min/1.73 m^2^ to 9% (95% CI, 4%–19%) at GFR of 30 mL/min/1.73 m^2^. In addition, among men anemia prevalence was 33% (95% CI, 11%–67%) at GFR of 15 mL/min/1.73 m^2^ and among women it was 67% (95% CI, 30%–90%) at GFR of 15 mL/min/1.73 m^2^.^[4]^ In a cross-sectional, US multicenter survey of 2004, the rate of anemia was much higher at 47.7% in patients with CKD.^[36]^ The percentage of patients with Hb ≤12 g/dL increased from 26.7% to 75.5% as GFR decreased from ≥60 mL/min/1.73 m^2^ to <15 mL/min/1.73 m^2^. Similar to the current study, even in the United States, the prevalence of anemia was strongly associated with declining GFR (8.4% at stage 1 to 53.4% at stage 5), and this trend has been reported by several other authors.^[8,37]^ In the current study, the prevalence of anemia in Chinese patients with CKD not receiving dialysis was 51.50%. The prevalence of anemia significantly increased with advancing CKD stage, stage 1: 22.4%, stage 2: 30.0%, stage 3: 51.1%, stage 4: 79.2%, and stage 5: 90.2%, suggesting the worsening situation in China. A systematic review by Jing et al in 2012 concluded that targeting low Hb levels is beneficial to patients with CKD, especially in the predialysis population; nevertheless, optimal Hb targets still remain elusive. Furthermore, a statistically significant increased risk of mortality was noted in the high Hb-level group (Relative risk (RR) 1.18; 95% CI, 1.02–1.37) among CKD patients with anemia; however, both the high and low Hb groups were treated with ESAs. High Hb levels demonstrated increased risk of hypertension (RR 1.40; 95% CI, 1.11–1.75), stroke (RR 1.73; 95% CI, 1.31–2.29), and hospitalizations (RR 1.07; 95% CI, 1.01–1.14) as compared with low Hb levels.^[38]^ The target level of Hb recommended by NKF in adult patients with CKD is 11 to 12 g/dL.^[13]^ A large-scale study across the European Union considered the target Hb at 10 to 12 g/dL and observed a 62% target-achieving rate in patients undergoing dialysis.^[39]^ In the current study, the target-achieving rate was considerably lower: 26.4% with a target of 10 to 12 g/dL and only 8.2% with a more aggressive target of 11 to 12 g/dL. These results suggest that anemia in patients with CKD in China needs to be addressed more proactively. The current observation might have an impact on the prescription pattern of antianemic drugs followed by physicians in China. This will help in designing an evidence-based algorithm for improving care of anemia in patients with ND-CKD.

The most frequent cause of ESRD in developed countries is diabetic nephropathy and hypertensive nephropathy.^[23]^ In China, glomerulonephritis is a major underlying cause of CKD.^[24]^ Moreover, as reported by a retrospective analysis of 2012 and few other studies, diabetes, hypertension, and interstitial nephritis have increased and become the leading causes of CKD in elderly Chinese patients.^[40–42]^ Similarly, in the current study, chronic glomerulonephritis (57.26%) was found to be the most frequent cause of CKD, followed by diabetic nephropathy and hypertensive renal damage. Different background diseases are associated with a distinct timing of anemia onset and severity. In diabetic patients, anemia seems to develop at an earlier stage of renal dysfunction. At any level of GFR, anemia is more frequent and severe in diabetic patients than in nondiabetic patients.^[43]^ In a 5-year prospective observational study conducted in a diabetes clinic in Australia, anemia was found in early kidney disease and anemia was more common among those with higher levels of albuminuria.^[44]^ The current study showed that patients with diabetic nephropathy are more likely to have anemia early in CKD and more likely to experience severe anemia. This is attributed to iron and EPO deficiencies, hyporesponsiveness to EPO, and hypoxia associated with diabetes.^[6,45]^

The 2007 NKF-KDOQI guidelines recommend considering the use of ESAs such as recombinant human EPO in adult ND-CKD patients with Hb levels <10 g/dL. Iron supplementation is another widely used treatment strategy. Analyzing the treatment pattern using ESA in China, approximately one-third of the anemic patients received treatment with EPO and/or iron products. Of the patients receiving EPO in the current study sample, the treatment was initiated at a Hb level of <8 g/dL in 41% of the patients and <7 g/dL in 22.7% of the patients, suggesting that the initiation of EPO treatment is late in Chinese patients with CKD. On the contrary, in the United States, it was found that 15% of medical trainees had initiated anemia treatment at Hb levels <11 g/dL.^[46]^ These observations reflect a less-than-optimal screening/monitoring/management of patients with CKD in China.

Transferrin saturation and serum ferritin level are the 2 indicators that are widely used to examine iron metabolism. The 2007 NKF-KDOQI guidelines recommend a target value of >100 ng/mL for serum ferritin in patients with ND-CKD and patients undergoing peritoneal dialysis and a target level of >20% for transferrin saturation in patients with CKD regardless of the dialysis status. In the current study, of the 1246 patients with anemia, 338 (27.1%) patients received iron products. Of these patients, 29.7% of the patients had a ferritin level of <100 ng/mL and 41% had a transferrin saturation of <20%. Of the 1246 patients, 245 (19.7%) received oral iron supplementation, whereas only 93 (7.5%) patients received the more effective and recommended intravenous iron supplementation. Suboptimal iron supplementation may partly explain why patients treated with EPO in the current study responded poorly, as suggested by previous studies.^[47,48]^ Overall, the reported treatment rates for both the treatment strategies were typically low in China. Similar findings were reported by DOPPS, according to which the target Hb achieved was low (39%–46%) in the 3 most developed metropolitan areas in China (Beijing, Shanghai, and Guangzhou), in addition to a low rate of intravenous iron use in patients undergoing hemodialysis: 41% in Beijing, 18% in Shanghai, and 45% in Guangzhou.^[22]^ On the contrary, in the United States, anemia treatment is mostly initiated within 3 months to 1 year of diagnosis of anemia in patients with CKD.^[8]^

This study reflects a nonpromising treatment pattern of anemia in patients with CKD in Shanghai, which is one of the most developed areas in China with the best medical resources and the most educated population, leading to the speculation of the worsening situation in the entire country.

To summarize, the prevalence of anemia in patients with ND-CKD is continuously increasing in China, with glomerulonephritis being the most common underlying condition for the development of CKD. There is a substantial gap between clinical practice and the guidelines. EPO and iron supplementation are not used in many patients who could have benefited from these treatments. Considering the economic status of Shanghai, data from the current study could be a gross underestimation of the problems in the entire country. In China, physician awareness of anemia in patients with ND-CKD is extremely essential to improve healthcare outcomes and QoL.

This study has certain limitations. Several elements of the study design may have an impact on the final outcome, and therefore findings must be interpreted with caution. The cross-sectional study design does not allow drawing true causal associations with certainty. Second, sampling was not done randomly. In addition, numbers of patients were limited in the study, and because the cohort is selected from 25 hospitals in Shanghai, the results cannot be generalized to the entire Chinese population. Another potential limitation is that there might be some variation in evaluating laboratory parameters despite following standardized protocol. Furthermore, it is reported that Hb levels may differ by sex, age, pregnancy, altitude, and smoking status. However, we referred the definitions of anemia given by the National Anemia Action Council and the World Health Organization for better clarity, and so did not consider the variables listed above. Our analysis of anemia treatment was restricted by the fact that NHANES data on the multiple treatment modalities for anemia (e.g., ESAs, iron) were scarce. Furthermore, well-designed, large-sample cohort studies and randomized clinical trials are recommended.

## 5 Conclusion

In conclusion, the current study reveals a high prevalence of anemia in Chinese patients with ND-CKD, which is more frequent at higher stages of CKD. It highlights the need to improve multiple aspects of CKD management, including early diagnosis and treatment of anemia, as well as appropriate health strategies in patients with ND-CKD to delay the progression of renal failure.
